# Effects of High Intensity Training and High Volume Training on Endothelial Microparticles and Angiogenic Growth Factors

**DOI:** 10.1371/journal.pone.0096024

**Published:** 2014-04-25

**Authors:** Patrick Wahl, Felix Jansen, Silvia Achtzehn, Theresa Schmitz, Wilhelm Bloch, Joachim Mester, Nikos Werner

**Affiliations:** 1 Institute of Training Science and Sport Informatics, German Sport University, Cologne, Cologne, Germany; 2 Department of Molecular and Cellular Sport Medicine, Institute of Cardiovascular Research and Sport Medicine, German Sport University Cologne, Cologne, Germany; 3 The German Research Centre of Elite Sport, German Sport University Cologne, Cologne, Germany; 4 Department of Internal Medicine II, Cardiology, Pneumology and Angiology, Medical Faculty, University of Bonn, Bonn, Germany; Bristol Heart Institute, University of Bristol, United Kingdom

## Abstract

**Aims:**

Endothelial microparticles (EMP) are complex vesicular structures shed from activated or apoptotic endothelial cells. As endurance exercise affects the endothelium, the objective of the study was to examine levels of EMP and angiogenic growth factors following different endurance exercise protocols.

**Methods:**

12 subjects performed 3 different endurance exercise protocols: 1. High volume training (HVT; 130 min at 55% peak power output (PPO); 2. 4×4 min at 95% PPO; 3. 4×30 sec all-out. EMPs were quantified using flow cytometry after staining platelet-poor-plasma. Events positive for Annexin-V and CD31, and negative for CD42b, were classified as EMPs. Vascular endothelial growth factor (VEGF), migratory inhibiting factor (MIF) and hepatocyte growth factor (HGF) were determined by ELISA technique. For all these measurements venous blood samples were taken pre, 0′, 30′, 60′ and 180′ after each intervention. Furthermore, in vitro experiments were performed to explore the effect of collected sera on target endothelial functions and MP uptake capacities.

**Results:**

VEGF and HGF significantly increased after HIT interventions. All three interventions caused a significant decrease in EMP levels post exercise compared to pre values. The sera taken after exercise increased the uptake of EMP in target endothelial cells compared to sera taken under resting conditions, which was shown to be phosphatidylserin-dependent. Increased EMP uptake was associated with an improved protection of target cells against apoptosis. Sera taken prior and after exercise promoted target endothelial cell migration, which was abrogated after inhibition of VEGF.

**Conclusion:**

Physical exercise leads to decreased EMP levels and promotes a phosphatidylserin-dependent uptake of EMP into target endothelial cells, which is associated with a protection of target cells against apoptosis.

## Introduction

In the past years there has been a lively discussion about high intensity training (HIT) and high volume training (HVT), their similarities and differences in adaptations. It was shown, that HIT is not only a useful tool in elite sports, but also in terms of health prevention and rehabilitation. Most studies focused on changes of endurance performance, muscle adaptations or health benefits, giving us a better idea of the molecular mechanisms [1], [2]. However, only a few studies focused on endothelial activation, function and adaptations in response to HIT and HVT. Interestingly, it seems that high intensities are needed to increase angiogenic growth factors (e.g. VEGF) [3] and to increase capillarization and endothelial cell (EC) proliferation [4], [5].

Vascular endothelial growth factor (VEGF), hepatocyte growth factor (HGF) and macrophage migration inhibitory factor (MIF) are angiogenic agents known to be involved in the regulation of growth of new blood vessels. These growth factors play a role in transcapillary permeability, matrix deposition and degradation and stimulate cell differentiation, proliferation, migration, and survival [6], [7]. For a long time, VEGF is known to be the most powerful inducer of angiogenesis. However, recent studies have shown HGF to be a powerful inducer of angiogenesis as well [8]. Furthermore, MIF also plays a role in promoting angiogenesis, for example by regulating cell proliferation and enhancing the production of VEGF [9]–[11]. Despite the knowledge of the angiogenic actions of the mentioned growth factors, little is known about the effects of exercise and especially about the effects of different training intensities on these factors.

In the context of angiogenic processes, endothelial microparticles (EMP) are becoming an upcoming marker of endothelial health. EMP are complex vesicular structures shed from activated or apoptotic endothelial cells. They are considered to play a remarkable role in coagulation, inflammation, endothelial function, and angiogenesis. Besides their role as marker of cell damage, recent reports have underlined their function as signalling elements in cell–cell communication. It has been reported that profiles of EMP reflect endothelial status and that EMP are generated under certain blood flow conditions (shear stress) and/or by a number of cytokines and apoptotic stimuli [12], [13]. As endurance exercise alters the blood flow velocity and therefore shear stress at the vascular wall, as well as the cytokine profile, the objective of the study was to examine changes in EMP following different endurance exercise protocols. Up to now, no study investigated the effects of different training intensities on EMP and only a few addressed the effects on angiogenic growth factors. The present study, therefore, focused on the effects of different exercise intensities on circulating VEGF, HGF and MIF concentrations and circulating EMP levels. It is hypothesized that higher exercise intensities lead to increases of angiogenic agents and to higher levels of EMP due to higher shear stress at the vascular wall.

## Methods

### Subjects

Twelve healthy, non-smoking male triathletes/cyclists (mean ± SD, age: 24.7±3.4 years, weight: 77.5±6.3 kg, height: 183.9±6.3 cm, relative VO_2_max: 64.3±9.7 ml·min^−1^·kg^−1^) participated in the present study. All subjects were informed orally and in writing of the study's purpose and the possible risks involved before providing written informed consent to participate. The study was approved by the Ethic Committee of the German Sport University Cologne in compliance with the Declaration of Helsinki. Subjects were assigned an anonymous ID during the study.

### Exercise study protocol

Before the participation, subjects performed a step test to determine maximal oxygen uptake (VO_2_max) (Zan 600, Zan Messgeräte, Oberthulba, Germany) and maximal peak power output (PPO) in order to determine the proper intensity for warming-up, recovery and the main training load (HVT & 4×4 min) for each subject for the main experiments. The step test consisted of cycling at ≥80 rpm with an initial workload of 100 W for 5 minutes and incremental 40-W increases every 5 minutes until volitional exhaustion was reached.

**Figure 1:**
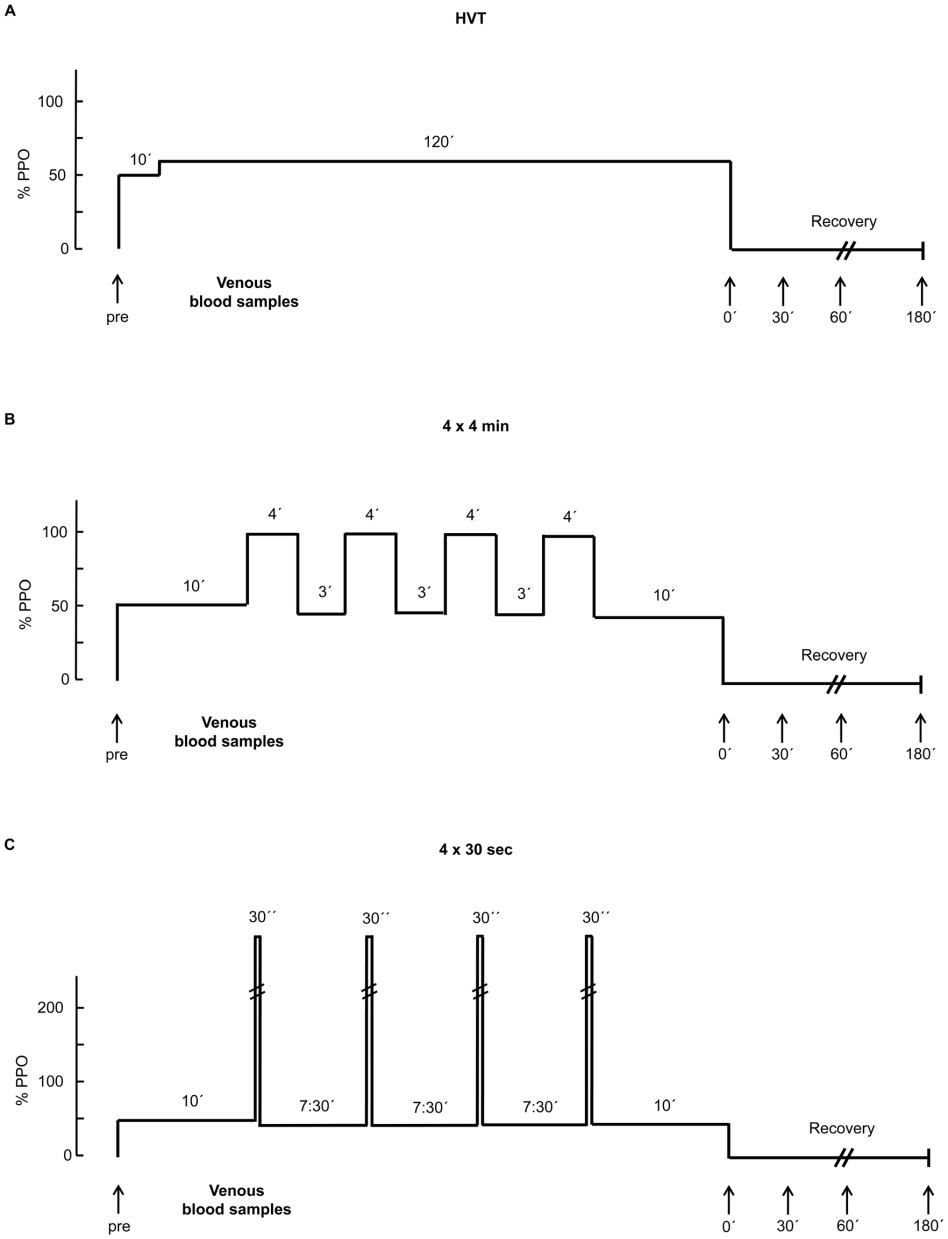
Exercise study protocols. The HVT protocol lasted a total of 130% of PPO followed by 120 min at 55% PPO (A). The 4×4 min protocol lasted a total of 45 min and consisted of a 10 min warming up at 50% of PPO followed by four 4 min intervals at 90–95% of PPO separated by 3 min active recovery (10 min recovery after the last bout) at 45% of PPO (B). The 4×30 sec protocol lasted a total of 44,5 min and consisted of a 10 min warming up at 50% of PPO followed by four 30 s all out exercise bouts separated by 7∶30 min active recovery (10 min recovery after the last bout) at 45% of PPO (C). Arrows assign time points for venous blood sampling. Venous blood samples for hormonal analysis were taken before exercise (pre), and four samples were taken at 0 min (0′), 30 min (30′), 60 min (60′) and 180 min (180′) post exercise.

To test the hypothesis subjects participated in three experimental trials on a cycle ergometer (Schoberer Rad Meβtechnik SRM GmbH, Jülich, Germany), each separated by one week in a randomized order (Figure 1 a–c). 1) “HVT”: 2 h at 55% of PPO; 2) “4×4 min”: 4×4 min at 90–95% PPO separated by 3 min of active recovery (45% PPO) each; 3) “4×30 sec”: 4×30 sec maximal effort (“all out”) separated by 7∶30 min of active recovery (45% PPO) each. For the “all-out” bouts the ergometer was adjusted to an isokinetic mode set to a cadence of 120 rpm. Subjects were instructed to perform the tests in a sitting position on the ergometer. During the all-out bouts all subjects were vocally encouraged to achieve maximal power output in the same way. For each 30 s all-out exercise bout, mean power output (MP) was calculated. Before each experimental trial, subjects warmed up for 10 min at an intensity of 50% PPO. During each session environmental conditions (temperature and humidity) were kept constant and all three tests were carried out at the same time of day in order to prevent diurnal variations in performance and the hormonal status. Always the same two investigators attended the tests. During each session spirometric data (VO_2_, VCO_2_, ventilation) were determined.

The food intake before the tests was partially standardized. That is, certain foods containing carbohydrates were recommended to the subjects, and the subjects were advised to ingest the same amount and composition of food before each test. Therefore, food intake was recorded before the first test and reproduced before the following. A last light meal was allowed 2 hours before the test. 30 minutes after each of the three tests, subjects received 500 ml of a low fat chocolate milk (6.5 g fat, 15 g protein, 46 g carbohydrate) and additional corn-bars according to their calculated energy expenditure of the trials. After the ingestion, subjects were only allowed to drink water until the last blood sample was withdrawn. The subjects were not allowed to perform strenuous exercise 24 hours before testing.

### Calculations

Total energy expenditure (EE) [kJ] and total work were determined for each session. Total EE was considered as the sum of EE during warm-up (EE_WU_), EE during the intervals (EE_Int_) and EE during the recovery periods (EE_Rec_). For each of the periods, EE was calculated separately according to Scott et al. [14] based on oxygen consumption and lactate production (Δlactate):
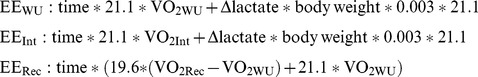



During warm-up and during the intervals, a caloric equivalent of 21.1 kJ per l oxygen was used, and for the recovery periods, the caloric equivalent of the excess post-exercise oxygen consumption was set at 19.6 kJ per l oxygen. An increase in 1 mmol/l of lactate was considered equivalent to 3 ml of oxygen per kg body weight[14]. For WU, Δlactate was defined as the difference between after WU- and rest-values, and for the intervals, Δlactate was equivalent to the difference between the value after the interval and after WU. The first interval was considered representative for all intervals so that the relative contribution of Δlactate was extrapolated based on the mean power during the interval. Total work [kJ] was calculated by the following formula: Total work  =  (MPO [W]·exercise time [s])·1000^−1^.

### Measurements

Venous blood samples were collected for the determination of vascular endothelial growth factor (VEGF), migratory inhibiting factor (MIF), and hepathocyte growth factor (HGF) before exercise (pre), and 0 min (0′), 30 min (30′), 60 min (60′) and 180 min (180′) post exercise (Figure 1 a–c). A venipuncture was performed for each sample. Nine and a half milliliters of blood was collected by the Vacutainer blood withdrawal system (Becton Dickinson). After storage at 7°C for ∼30 min for deactivation of coagulation factors, the blood samples were centrifuged for 10 min at 1.861 g and 4°C (Rotixa 50, Hettich Zentrifugen, Mühlheim, Germany). The serum was stored at −80°C till analysis. Serum levels of VEGF (pg·mL^−1^), MIF (ng·mL^−1^) and HGF (pg·mL^−1^) were determined by using human ELISA kits for VEGF (Human VEGF Quantikine ELISA Kit (Catalog Number DVE00)), MIF (Human MIF Quantikine ELISA Kit (Catalog Number DMF00B), and HGF (Human HGF Quantikine ELISA Kit (Catalog Number DHG00)) R&D Systems, Inc. USA. All results of the growth factor analysis were adjusted for changes in plasma volume (PV) [PV changes in percentage of pre values  =  [(Hb_pre_/Hb_post_)•(100-Hct_post_)/[(100-Hct_pre_)-1]]•100 [15].

For the determination of EMP, monocytic micropartilces (MMP) and platelet-derived microparticles (PMP) 8 mL of heparin blood was withdrawn pre, 0′, 60′ and 180′. EMPs were quantified (number/μl) using flow cytometry after staining platelet-poor-plasma with the cell surface markers. Events positive for Annexin-V and CD31, and negative for CD42b, were classified as EMPs. For more detailed information see [16], [17]. Events positive for Annexin-V, CD31 and CD42b were classified as PMP, events positive for Annexin-V, CD14 and CD16 were classified as MMP.

During each of the three sessions capillary samples from the earlobe were collected for blood gas (AVL Omni 6; Roche Diagnostics GmbH, Mannheim, Germany) and lactate analysis (EBIOplus; EKF Diagnostic Sales, Magdeburg, Germany) at the following time points during each intervention:

1) HVT: Blood gas/lactate: resting condition, post WU, and every 30 min during exercise.

2) 4×4 min: Blood gas: resting condition, post WU, and 30 sec/2∶30 min after each 4 min exercise bout. Lactate: resting condition, post WU, and 0 sec/3 min after each 4 min exercise bout.

3) 4×30 sec: Blood gas: resting condition, post WU, and 30 sec/6∶30 min after each 30 sec exercise bout. Lactate: resting condition, post WU, and 0 sec/3∶30 min/7 min after each 30 sec exercise bout.

For the comparison of lactate and blood gas values of the 3 interventions the mean of all measurements and all subjects during each intervention was calculated.

### In-vitro experiments

#### EMP uptake

The collected sera from 3 randomly chosen athletes of different conditions (4×4 and 4×30) were used for ex vivo stimulation of human coronary artery endothelial cells (HCAEC). HCAEC were incubated for 4 h (37°C) with 10% of the conditioned serum samples (900 µl growth factor deprived medium + 100 µl of athlete's serum of the following time points: pre, 0′, and 180′. Afterwards, serum was removed and the cells were washed with sterile PBS 3 times. To explore whether stimulation of HCAEC with sera influences the uptake of EMP, 5×10^5^ EMP were generated from starved HCAEC, labeled with PKH26 and diluted in 1 mL PBS as previously described [18]. After 4 h incubation at 37°C, HCAEC were washed extensively with PBS and fixed with 2% PFA for 10 min at 37°C. Blocking was performed with 0.5% bovine serum albumin (BSA) for 30 min. The primary antibodies used was mouse-anti-human PECAM-1 (1∶50; Sigma Aldrich), which was diluted in 0.5% BSA-PBS and incubated overnight at 4°C. The secondary antibody was rat-anti-mouse IgG-Cy2 (1∶200, Sigma Aldrich), which was incubated for 1 h at room temperature. After staining using mounting medium containing DAPI (Vectashield, Vector Laboratories, Burlingame, California, USA) the total number of EMPs per cell were counted.

For MMP uptake experiments, MMP were generated from THP-1 cells and labelled with calcein as previously described [19]. Uptake experiments were performed as described for EMP.

To explore whether annexin V binding to phosphatidylserin (PS) modulates EMP uptake, EMPs were preincubated with or without annexin V (3 µg/mL) for 1 hour in the presence of CaCl_2_ (5 mmol/L final concentration) as previously described by Rautou et al. [20] before uptake experiments were performed as described.

#### Apoptosis

Apoptosis was assessed using Caspase-3 ELISA as previously described [21]. Briefly, confluent HCAEC in 24 well plates were pretreated with EMP and serum for 20 hours and then subjected to 1 µM camptothecin for 4 hours to induce apoptosis. Next, cells were washed with sterile PBS and lysed in 150 µl of CHAPS lysis buffer (10 mM HEPES, pH 7.4, 42 mM KCl, 5 mM MgCl2, 0.1 mM EDTA, 0.1 mM EGTA, 1 mM PMSF, 1 mM DTT, 0.5% CHAPS, Roche Complete Mini protease inhibitors according to the manufacturer's instructions) on ice. 50 µl of lysate was incubated in 150 µl reaction buffer (25 mM HEPES, pH 7.5, 1 mM EDTA, 0.1% CHAPS, 10% sucrose, 3 mM DTT) and supplemented with 10 µM of the specific fluorogenic caspase 3 substrate Ac-DEVD-AMC (Bachem) for 2 hours at room temperature. Accumulation of fluorescent AMC was measured at 385 nm (excitation) and 465 nm (emission) using a fluorescence 96-well plate reader. Detected fluorescent signals were normalized for protein content, as determined by Lowry protein assays (Biorad).

#### Migration and proliferation

Migration and proliferation were assessed using scratch assay as previously described [18]. In brief, endothelial cells were grown to confluence, treated with sera and scratched with a sterile pipette (200 ul). To explore the role of VEGF and HGF, we stimulated target cells additionally to serum incubation with the VEGF inhibitor sunitib malate (Selleckchem, Houston, US) and the HGF inhibitor PHA665752 (Selleckchem, Houston, US) as previously described [22], [23] according to the protocol provided by the company. Cells were photographed on a marked position at 0, 6 and 20 hours. Zeiss Axiovert 200 M microscope and AxioVision software were used to take photographs.

### Statistics

Statistical analyses of the data were performed by using a statistics software package (Statistica for Windows, 7.0, Statsoft, Tulsa, OK). Descriptive statistics of the data are presented as means ± SD, unless described otherwise. All data were normally distributed (Shapiro-Wilk test). To assess the effect of the three different interventions on circulating angiogenic growth factors and EMP, a 2-factor [intervention (4×30 sec, 4×4 min, HVT); time (pre-, 0′, 30′, 60′, 180′)] repeated-measures ANOVA with Fisher post-hoc test was used. For each growth factor/EMP we reported the *p*-value corresponding to the main intervention effect and time effect. Statistical differences were considered to be significant for p<0.05. Furthermore, the effect size “partial η^2^” was calculated according to the main effect over time for each intervention and parameter. The thresholds for small, moderate, and large effects were defined as 0.1, 0.25 and 0.4, respectively.

## Results

### Power output, total work, oxygen uptake, RER, lactate and blood gas analysis

In order to characterize the three interventions, the results for the mean power (MP) output, mean lactate concentrations [La], mean pH, mean base excess (BE), mean pO_2_, mean pCO_2_, total work, energy expenditure, total oxygen consumption, heart rate (HR) and rate of perceived exertion (RPE) for all three interventions are shown in Table 1. Therefore, the mean of all measurements and all subjects during one intervention was calculated. All parameters differed significantly from each other between the three interventions. Only HR and RPE did not differ between the two HIT interventions.

**Table 1:** Overview of the three exercise interventions.

	4×30 sec	4×4 min	HVT
**Total time [min]**	44.5	45	130
**Intensity [%PPO]**	All-out	90-95	55
**Mean power [W]**	657±54	278±39*	164±23* ^#^
**Mean [La] [mmol•L^−1^]**	8.8±2.0	4.4±1.7*	1.0±0.2* ^#^
**Peak [La] [mmol•L^−1^]**	12.8±2.4	6.6±2.5	1.2±0.4
**Mean pH [AU]**	7.26±0.04	7.34±0.03*	7.39±0.01* ^#^
**Min pH [AU]**	7.19±0.06	7.31±0.04	7.39±0.01
**BE [mmol•L^−1^]**	−10.6±2.4	−6.0±2.1*	−1.2±0.9* ^#^
**pO_2_ [mmHg]**	87.9±6.5	82.8±5.3*	77.0±5.5* ^#^
**pCO_2_ [mmHg]**	34.5±3.8	36.7±2.2*	39.4±1.7* ^#^
**Total work [kJ]**	430±58	509±71*	1219±173* ^#^
**Energy expenditure [kJ]**	2439±354	2842±326*	6152±757* ^#^
**Total oxygen consumption [L]**	118±18	134±15*	291±36* ^#^
**RPE [AU]**	18.3±1.2	16.0±1.2	12.3±1.5* ^#^
**HR [bpm]**	160±12	155±11	138±13* ^#^
**Peak HR [bpm]**	180±11	176±14	138±15

[La] = lactate concentration; BE = base excess; pO_2_ = partial pressure of oxygen; pCO_2_ = partial pressure of carbon dioxide; HR = heart rate; RPE = rate of perceived exertion. For the “mean values” the mean of all measurements and all subjects during each intervention was calculated. Data are shown as mean ± SD. * significantly different from “4×30 sec”; ^#^ significantly different from “4×4 min”.

### VEGF

**Figure 2:**
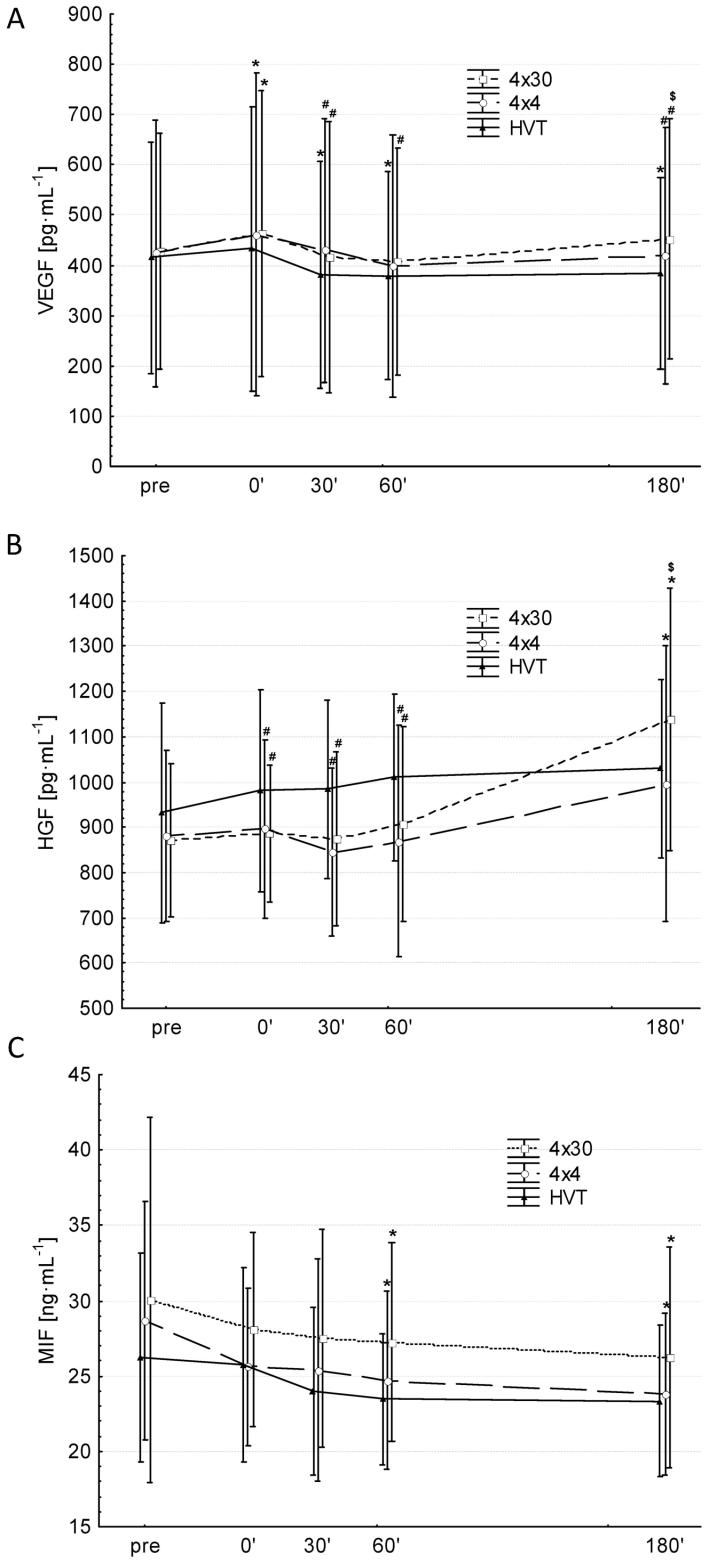
Changes in circulating VEGF (A), HGF (B) and MIF (C). 4×30 sec (Squares, dotted line), 4×4 min (circles, broken line) and HVT (triangles, solid line). * significantly different compared to pre-values (p≤0.05). Values are presented as means ± SD.

VEGF showed large inter-individual differences ranging from 140 pg•mL^−1^ up to 834 pg•mL^−1^ (min – max of all pre-values). Post-hoc analysis revealed that both HIT interventions (4×30 sec & 4×4 min) significantly increased VEGF levels 0′ after exercise (p = 0.004 & p = 0.006) compared to pre-values. However, VEGF levels decreased back to pre values 30′ after the exercise (Figure 2a). HVT significantly decreased VEGF levels 30′, 60′ and 180′ compared to pre values. Compared to HVT, 4×30 sec showed higher values at 30′, 60′ and 180′ and 4×4 min at 30′ and 180′ post intervention. Compared to 4×4 min, 4×30 sec showed higher VEGF values 180′ post intervention (both p<0.001). Values for the effect size “Partial η^2”^ are given in table 2.

**Table 2:** Values for the effect size “partial η^2^”.

	4×30 sec	4×4 min	HVT
**VEGF**	0.23	0.22	0.22
**HGF**	0.50	0.22	0.10
**MIF**	0.07	0.24	0.11
**EMP**	0.30	0.39	0.25
**MMP**	0.29	0.31	0.12
**PMP**	0.54	0.26	0.36

The thresholds for small, moderate, and large effects were defined as 0.1, 0.25 and 0.4, respectively.

### HGF

Post-hoc analysis revealed that both HIT interventions (4×30 sec & 4×4 min) significantly increased HGF levels 180′ after exercise (p<0.001 & p = 0.01) compared to pre-values (Figure 2b). HVT did not significantly change HGF levels. Compared to HVT, 4×30 sec and 4×4 min showed significantly lower values 0′, 30′, 60′ post intervention. 180′ post exercise 4×30 sec showed significantly higher values compared to 4×4 min. Values for the effect size “Partial η^2^” are given in table 2.

### MIF

Post-hoc analysis revealed that both HIT interventions (4×30 sec & 4×4 min) significantly decreased MIF levels 60′ (p = 0.05 & p = 0.02) and 180′ after exercise (p = 0.03 & p = 0.004) compared to pre-values (Figure 2c). However, for HVT values nearly reached statistical significance 60′ & 180′ compared to pre-values (p = 0.08 & p = 0.09). Values for the effect size “Partial η^2^” are given in table 2.

In summary, HIT seems to stimulate a transient increase in circulating levels of VEGF and HGF but a decrease of MIF.

### Microparticle analysis

**Figure 3:**
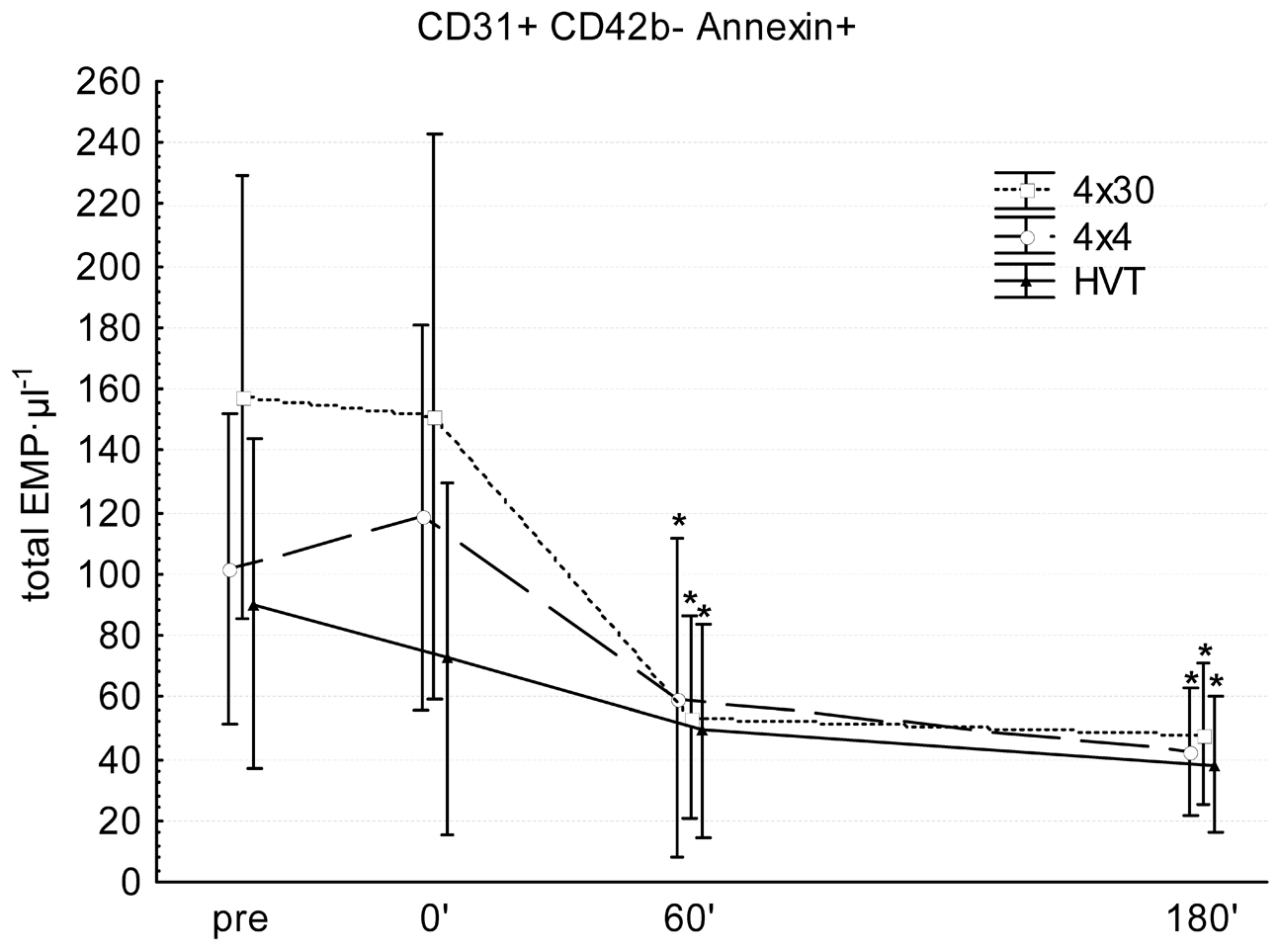
Changes in circulating EMP in conditioned serum of athletes. 4×30 sec (Squares, dotted line), 4×4 min (circles, broken line) and HVT (triangles, solid line). * significantly different compared to pre-values (p≤0.05). Values are presented as means ± SD.

For EMP over-all ANOVA showed significant differences for different points in time of one condition (p<0.0001; F = 8.84) but not for different conditions (p = 0.75; F = 0.29). Post-hoc analysis revealed that all three interventions (4×30 sec, 4×4 min and HVT) significantly decreased EMP levels 60′ (p = 0.05, p<0.001 and p = 0.01) and 180′ (p = 0.01, p<0.001 and p = 0.004) after exercise compared to pre-values (Figure 3). Values for the effect size “Partial η^2^” are given in table 2.

For MMP over-all ANOVA showed significant differences for different points in time of one condition (p<0.02; F = 4.25) but not for different conditions (p = 0.27; F = 1.44). Post-hoc analysis revealed that both interval protocols (4×30 sec, 4×4 min) significantly decreased MMP levels 60′ (p = 0.003 and p = 0.04) and 180′ (p = 0.003 and p = 0.004) after exercise compared to pre-values (figure S1A). Values for the effect size “Partial η^2^” are given in table 2.

For PMP over-all ANOVA showed significant differences for different points in time of one condition (p<0.0008; F = 7.85) but not for different conditions (p = 0.19; F = 1.82). Post-hoc analysis revealed that all three interventions (4×30 sec, 4×4 min and HVT) significantly decreased PMP levels 60′ (p<0.001, p = 0.02 and p = 0.003) and 180′ (all p<0.0001) after exercise compared to pre-values (figure S1B). Values for the effect size “Partial η^2^” are given in table 2.

As we previously studied the role of EMP in vascular biology in detail, [16], [18], [21], [24], we next aimed to explore potential mechanisms mediating the decrease of EMP levels after physical exercise.

### EMP uptake

**Figure 4:**
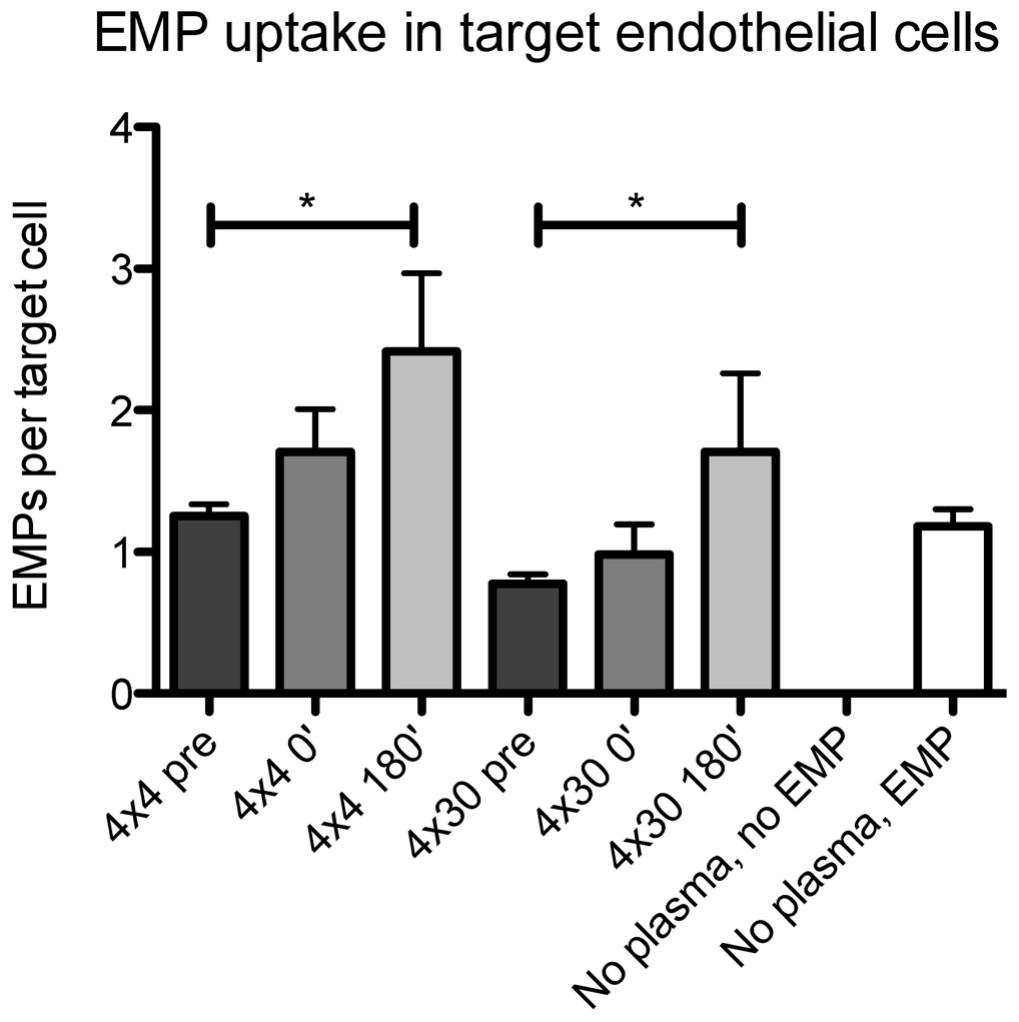
Uptake of EMP into target endothelial cells. HCAEC were stimulated with EMP and sera for 4>3, * = p<0.05.

To analyze whether the decrease of circulating EMP levels after physical exercise is mediated by an increased capacity of the endothelium to take up EMP, HCAEC were incubated with sera collected prior and after exercise (pre, 0′ and 180′). After incubation, HCAEC were treated with PKH26-labelled EMP for four hours and the number EMP per cell were analyzed. Interestingly, The sera collected 180′ after two different exercise protocols (4×4 min and 4×30 s) significantly promoted the uptake of EMP into target HCAEC compared to sera taken under resting conditions (pre) (p = <0.05, n>3, figure 4). These data suggest, that sera after physical exercise stimulate endothelial cells to take up EMP. Interestingly, stimulation of target cells with sera collected after exercise did not influence the number of uptaken MMP, suggesting that there are different clearing mechanisms between microparticles derived from distinct cells (figure S2).

**Figure 5:**
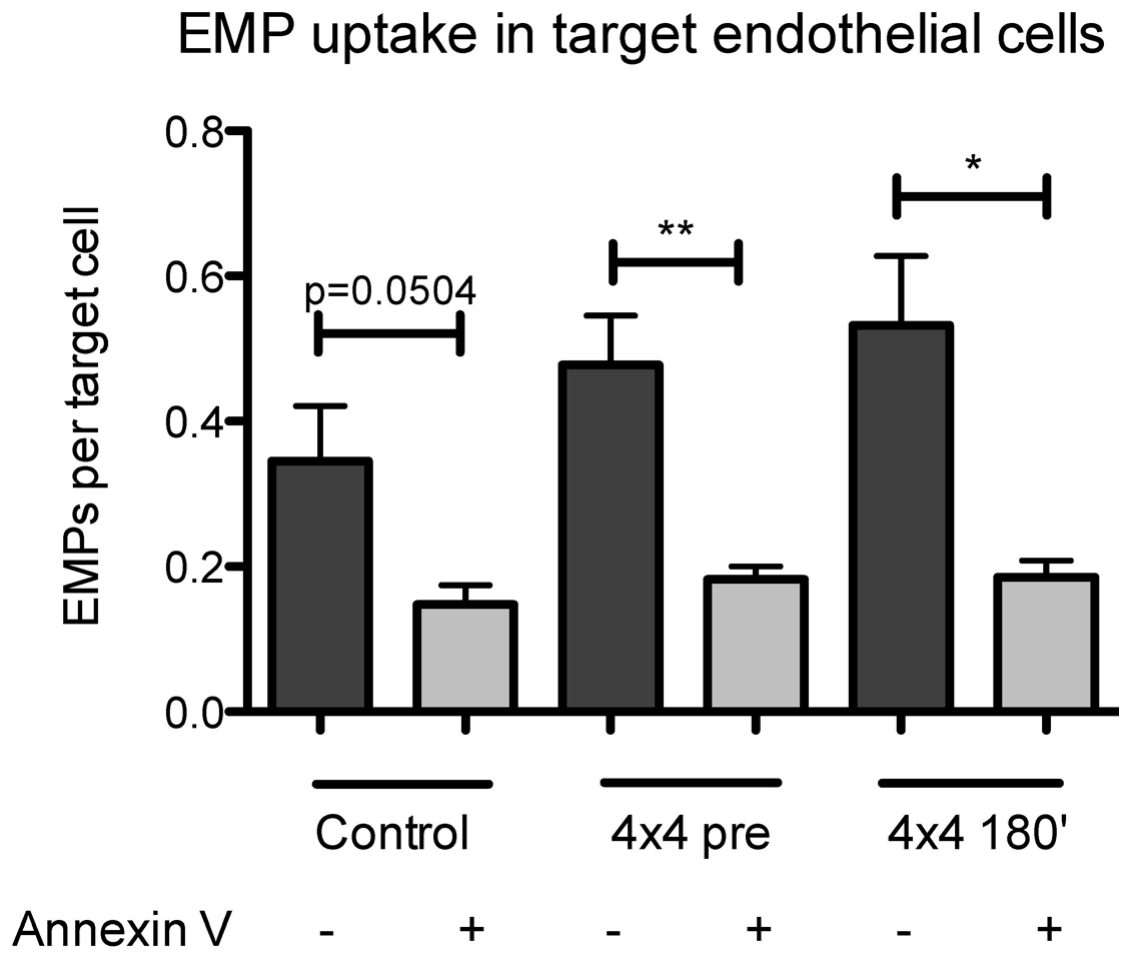
Annexin I prevents EMP uptake into target endothelial cells. HCAEC were stimulated with EMP and sera for 4(3 µg/mL) for 1 hour in the presence of CaCl2 (5 mmol/L final concentration). N = 5, * = p<0.5, ** = p<0.01.

Previously, we demonstrated that the uptake of EMP into target cells is annexin I-phosphatidylserinreceptor (PSR)-dependent [21]. We therefore next aimed to assess whether blockade of phosphatidylserin (PS) on EMP might alter EMP uptake into target HCAEC after stimulating HCAEC with sera collected prior (pre) and after (180′) exercise. Treatment of EMP with PS-binding annexin V significantly reduced the number of EMP taken up by endothelial target cells prior (p<0.01, n = 5, figure 5) and after exercise (p<0.05, n = 5, figure 5), suggesting that PS on EMP is crucially involved in EMP uptake mechanisms in target HCAEC.

### Functional experiments

**Figure 6:**
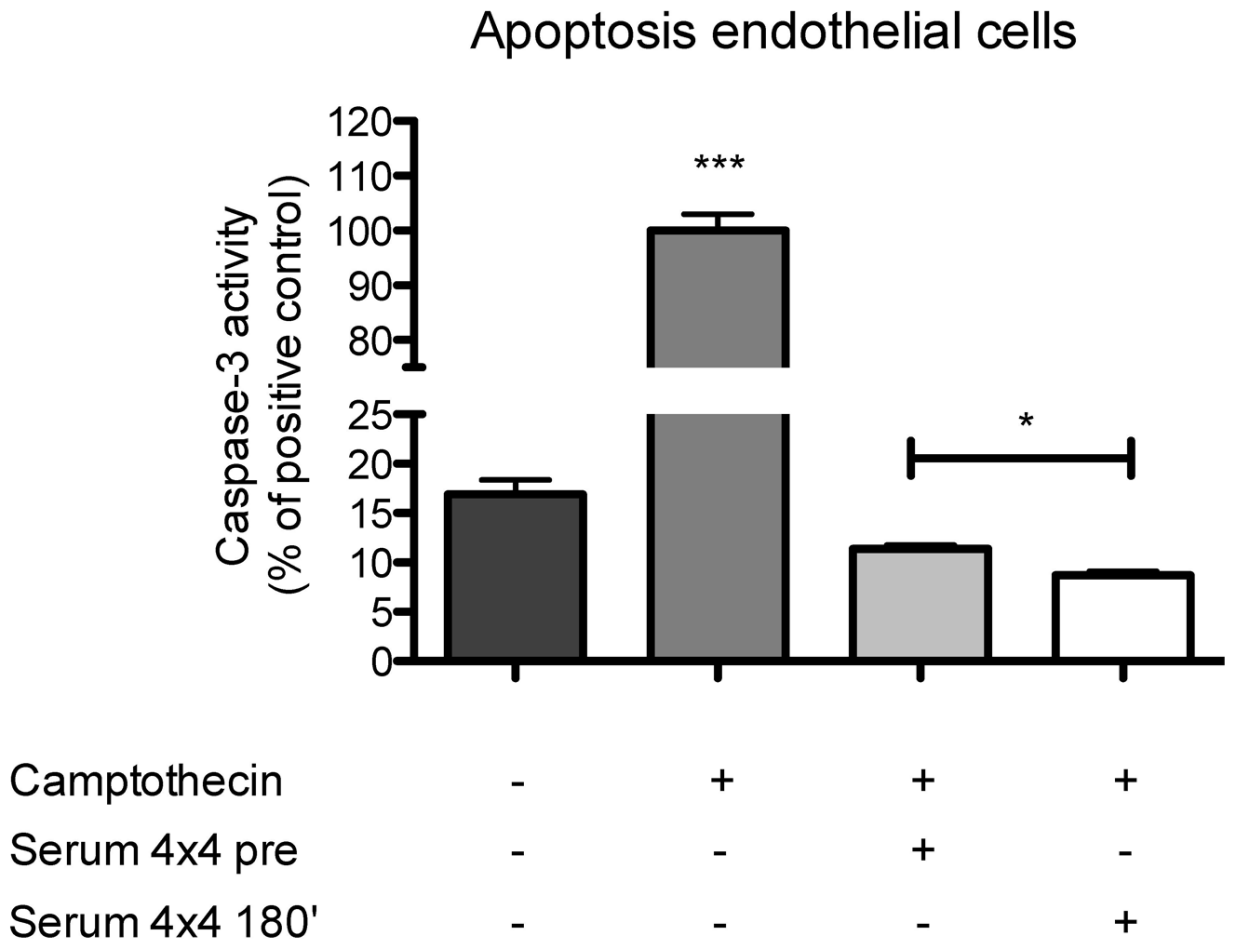
HIT promotes protection of endothelial target cell from apoptosis. HCAEC were pretreated with EMP and serum for 20 µM camptothecin for 4 hours to induce apoptosis. Caspase-3 activity of the positive control is significantly increased against all other groups. N = 5–10, * = p<0.05.

As EMP have been shown to protect target HCAEC from apoptosis [21], we next explored whether increased EMP uptake can modulate apoptosis in target cells. Therefore, HCAEC were treated with EMP and sera collected prior (4×4, pre) and after (4×4 180′) exercise and apoptosis was assessed detecting caspase-3 activity. Interestingly, stimulation of HCAEC with EMP and sera after exercise was associated with an improved protection against apoptosis compared to treatment with EMP and sera collected before exercise (n = 5–10, p<0.05, figure 6).

In order to explore whether sera collected prior and after exercise might modulate other biological functions of target cells in addition to apoptosis, we stimulated HCAEC with sera (4×4 pre and 180′) and assessed migration and proliferation capacities of target cells using scratch assay. Both conditions significantly promoted migration of target cells after 6 and 20 hours (figure S3A,B). However, there was no difference between cells stimulated with sera prior and after exercise. Since VEGF and HGF promote endothelial cell migration and proliferation and we found a transient increase in circulating levels of VEGF and HGF after HIT, we tested the effects of VEGF- and HGF inhibitors on sera-induced endothelial target cell migration. After six and twenty hours, inhibition of VEGF significantly reduced sera-induced endothelial cell migration (figure S3A,B). HGF inhibition did not influence target cell migration besides a decreased endothelial cell migration after six hours and treatment with sera 180′. However, treatment of target cells with VEGF- and HGF inhibitors further reduced target cell migration compared to VEGF inhibition alone, suggesting that the depletion of VEGF activity might uncover an anti-migratory effect of the HGF-inhibition on target cells (figure S3A,B).

## Discussion

The present study demonstrates that high intensity training (4×30 and 4×4) stimulates a transient increase in circulating levels of VEGF and HGF and a decrease of MIF, whereas HVT training had no influence on VEGF, HGF and MIF, despite a much higher training volume and time commitment (by design, the absolute exercise time was −65% lower in both HIT interventions (45 vs. 130 min) compared to HVT). Metabolic stress, characterized by values of lactate and blood gas measurements (pH, BE, HCO_3_^−^, pO_2_, PCO_2_) was highest during and after 4×30 sec, followed by 4×4 min and HVT. In contrast, total work, total energy expenditure and total oxygen consumption were ∼2/∼3times higher in HVT compared to 4×4 min and 4×30 sec. Furthermore all three training interventions acutely decreased the circulating EMP, MMP and PMP levels. In accordance with these results, incubated HCAEC showed an increased uptake of EMP after stimulation with conditioned serum samples of athletes, whereas MMP uptake was not influenced after serum stimulation of target cells. These findings suggest that microparticles derived from different cell types experience different clearance mechanisms. In this context, we and others demonstrated that the endothelium in vivo might be a potential locus for circulating microparticle uptake [18], [25]. Moreover, previous studies highlight the importance of spleen and liver macrophages as major determinants for the clearance of circulating microparticles from erythrocyte, platelet and endothelial cell origin [26], [27]. Taken together, the locus and the mechanisms of microparticle clearance in vivo are still fairly unclear and warrant further investigation [28].

In terms of a decrease in EMP, the present study supports previous findings, that short-term high intensity exercise elicits similar results than HVT. Although a few other studies have investigated microparticles following exercise in humans, this has been the first to examine EMP, MIF and HGF after different endurance training regimes.

The findings of studies investigating the VEGF response to exercise are heterogeneous [3], [29]–[31]. However, summing up these results, it seems that the VEGF response is dependent on the exercise intensity. High-volume low-intensity exercise causes no changes or a decrease in circulating VEGF levels. On the other hand, higher intensities cause an increase in VEGF. Therefore, the results for VEGF of the present study, showing increases of 8.4% (4×30 sec) and 8.7% (4×4 min) and no changes/a decrease for HVT are in line with the literature. However, Morici et al. (2005) showed larger increases of (+28%) after all out rowing, compared to the all-out exercise used in this study [31]. It can be speculated, that the major stimuli for a VEGF release/synthesis (hypoxia and shear stress) are more present during higher intensities than during HVT. In this case, it can be postulated that HIT is superior to HVT in inducing high circulating VEGF levels. Furthermore, previous studies suggested that microenvironment signals, such as acidosis and lactate play a major role in the control of VEGF production, and consequently in modulation of angiogenesis [32], [33]. These findings are generally in line with the present study, as pH values were lowest in 4×30 sec followed by 4×4 min and no changes during HVT. As the extracellular pH has been recognized as a regulator of the VEGF interactions with different cells, this might be an explanation for different VEGF levels after the three training interventions [34]. Previous studies showed that the decrease in plasma VEGF levels after HVT is not related to a peripheral uptake of VEGF to the exercising muscles [35]. Therefore, an uptake in other tissues, changes in binding properties of VEGF to plasma proteins, or a decreased release into the circulation during exercise may provide alternative explanations. The degree of uptake or release may depend on exercise intensity and duration, explaining the differences between HIT and HVT. In general, our findings suggest a potential venue to explore the mechanism underlying the higher gains in capillarity with HIT compared to HVT [36]. Similar to previous results [3], we also found high inter-individual differences in VEGF levels even for resting values.

Recent studies showed that HGF is a potent angiogenic factor in addition to VEGF regulating angiogenesis, vascular permeability, cell migration, matrix deposition and degradation [7], [37]. In contrast to the results of Rehman et al. (2004) who showed a non-significant increase of HGF of 8.8% [38], we were able to show significant increases of HGF after both HIT-interventions (30.7% (4×30 sec), 13.1% (4×4 min)) and a similar, but non-significant increase of 10,5% after HVT. However Rehman et al. (2004) used patients and a symptom-limited treadmill or bicycle exercise test which might explain the lower increases in their study. Similar to the present results, Morici et al. (2005) showed increases of + 41% after 1000 m all-out rowing [31]. In contrast to VEGF, HGF did not increase directly after exercise, but 180′ after the cessation of the intense training protocols. It can be speculated, that this increase might be due to a synthesis of HGF, whereas VEGF was acutely released by mechanisms explained previously. As no data are available about the inducing stimuli on HGF it can only be postulated, that HIT is superior to HVT in inducing high levels of HGF, may be favoring pro-angiogenic conditions.

MIF also has a role in promoting angiogenic processes [10]. Endothelial tube formation was augmented when endothelial cells were cultured with conditioned media from MIF-pretreated mononuclear cells [11]. Furthermore the production of the angiogenic factors VEGF and IL-8 was enhanced in cultured fibroblasts stimulated by MIF [11]. This might be due to a MIF mediated activation of HIF-1α under hypoxic conditions, which is known to regulate VEGF [39]. Neutralization of MIF by anti-MIF antibodies inhibited endothelial cell growth and led to a reduced number of tumor capillaries [9]. Despite the *in-vitro* data, only a few *in-vivo* data are available. In accordance to the results of Schmidt et al. (2009), MIF showed a significant decrease after high intensity exercise in the present study (−12,6% (4×30 sec), −17,1% (4×4 min)), which might favour the migratory activity of endothelial cells [40]. In their study, athletes exercised to exhaustion within a maximum time of 8 minutes, which caused a decreased in MIF levels of ∼20% [40]. In contrast, this decrease was already present directly after exercise, whereas in the present study, MIF-levels showed the first significant decreased after 60′. However, in their study an array kit was used to analyze MIF. The decrease of MIF after exercise might be explained by an uptake of MIF in the vascular wall, regulating local inflammatory processes, promoting the directed migration and recruitment of leukocytes into inflammatory sites [10]. This uptake might have a protective function for the vascular system.

As increases in microparticles were described following cell activation or apoptosis and in diseases with altered shear forces in blood vessels, we expected circulating EMP to be increased after endurance exercise, especially after higher intensities, as exercise is known to increase the blood flow/shear stress at the vascular wall. However, circulating EMP significantly decreased after all three exercise interventions in the present study. Although a few other studies have investigated EMP following exercise in humans, this has been the first to examine EMP after different endurance training regimes. The findings of studies investigating the acute response of circulating EMP to exercise are heterogeneous. No changes in circulating EMP concentrations were observed after 100 min exercise at 70% *V*O_2_peak [41] and after three separate progressive maximal ramp tests [42]. Increases, with maximum values at 45 min after exercise and returned basal levels 2 h after exercise, were observed after 90 min of bicycle ergometer exercise at 80% of the individual anaerobic threshold [43]. The observed decrease of the present study might be due to an increased clearance from the circulation, possibly mediated by an increased uptake from target endothelial cells. In accordance with data from Rautou et al. [20], we found that EMP are taken up in a phosphatidylserin-dependent manner by target endothelial cells. However, we cannot exclude an uptake of EMP to other types of cells or that increased EMP uptake under exercise conditions is mediated by qualitative changes of EMP released in this conditions. It has been shown that endothelial cells can also incorporate platelet-derived microparticles by active endocytosis modifying endothelial cell phenotype and function [44] and promoting angiogenesis [45].

Importantly, we found that increased uptake of EMP was associated with an improved protection of endothelial target cells against apoptosis. These findings are in accordance with previous data, showing that EMP protect target cells from apoptosis in an annexin I- phosphatidylserinreceptor-dependent manner [21]. Whereas apoptosis experiments revealed reduced apoptosis of target cells after treatment with serum after exercise compared to control serum, serum collected after physical exercise did not modulate migration capacities of endothelial target cells compared to control serum. But, interestingly, both serum conditions (pre and 180′) extensively promoted endothelial cell migration. The impressive induction of migration in both groups might overlie potential differences between these groups.

The influence of sera on the uptake of EMP to endothelial cells could be mediated by soluble factors. As the present study showed, physical activity up-regulates angiogenic growth factors (VEGF), which are pro-angiogenic and inhibit apoptosis of endothelial cells (EC) on the one hand, but simultaneously induces the expression of developmentally regulated endothelial cell locus 1 (DEL-1) [46], which was shown to mediate phosphatidylserine- and integrin-dependent endothelial uptake of microparticles by endocytosis [25]. In this context, one could speculate that the increase of VEGF and the decrease of circulating EMP after physical exercise might be mediated by an enhanced expression of DEL-1 with a subsequently increased uptake of EMP into the endothelium.

However, it should be recognized that baseline values (pre) showed great inter-individual differences, which is a clear limitations of the present study. Even though not statistically different, pre-values for 4×30 sec were higher compared to both other interventions. Nevertheless, it can be speculated that the combination of increased angiogenic growth factors and an increased EMP uptake protects the vascular system against apoptosis, despite massive stress. Additionally, low shear stress associates with increased EMP levels, suggesting that physiological shear stress like during exercise, known to contribute to endothelial survival, may limit EMP release [47].

Furthermore, the reduction of EMP in the circulation might lead to pro-angiogenic conditions. In particular, *in-vitro* studies showed that low concentrations of EMP could promote angiogenesis, whereas high concentrations could suppress angiogenesis [48], [49]. Low concentrations of EMP have been reported as pro-angiogenic through the matrix metalloproteinase activity that they harbour [13]. In sharp contrast, high concentrations of EMP have been reported as anti-angiogenic, as they decrease the formation of capillary-like structure by the production of reactive oxygen species (ROS) [49].

## Conclusion

Based on the data of VEGF, HGF and EMP it appears that especially intense training protocols may promote pro-angiogenic conditions. It can be speculated, that the acute hormonal increases and metabolic perturbations, might play a positive role in optimizing training adaptations. The described hormonal reactions may contribute to the positive effects of HIT even in highly trained athletes, as it has been shown by previous studies using similar exercise protocols. Finally, strenuous physical activity leads to an increased uptake of EMP which might protect EC and therefore the whole vascular system against apoptosis. This could be a mechanism how the endothelium is protected against distinct damage, despite massive stressors like high shear stress and/or decreases in pH. Although we tested well trained athletes in the present study, the results might also be important for therapeutic strategies. Our results may lead to the assumption, that the shear stress induced by exercise has positive effects in contrast to the shear stress in certain pathological conditions. In terms of a decrease in circulating EMP, the present study supports previous findings, that short-term high intensity exercise elicits similar results than HVT. As Mezentsev et al. 2005 stated, future therapeutic strategies aimed at reducing the number of circulating endothelium-derived microparticles or blocking their effects may be reasonable in different pathological processes where abnormalities of neovascularization and angiogenesis prevail [49]. Therefore, future studies should focus on the observed decreasing effects of exercise on EMP in other populations. Furthermore, a quantitative investigation of the microparticles and their content should be focused in order to provide better mechanistic insight.

## Supporting Information

Figure S1 Changes in circulating MMP and PMP in conditioned serum of athletes. 4×30 sec (Squares, dotted line), 4×4 min (circles, broken line) and HVT (triangles, solid line). * significantly different compared to pre-values (p≤0.05). Values are presented as means ± SD.
(TIFF)

Figure S2 Uptake of MMP into target endothelial cells. HCAEC were stimulated with MMP and sera for 4 hours. N>3.
(TIFF)

Figure S3 Migration of endothelial cells after stimulation with conditioned sera. HCAEC were stimulated with sera and with our without VEGF and HGF inhibitors. HAEC pretreated with growth factor deprived medium served as control. Migration was assessed after six (A) and twenty (B) hours. N = 5. A: ** = p<0.01 vs. Control, pre/180′ + VEGF-inhibitor and 180′ + HGF-inhibitor, #  =  p<0.001 vs. all other groups except control. B: *** = p<0.001 vs. Control and pre/180′ + VEGF-inhibitor, #  =  p<0.001 vs. all other groups except control.
(TIFF)
