# Supplementary figures and images for: Effects of High Intensity Training and High Volume Training on Endothelial Microparticles and Angiogenic Growth Factors
Source: PLoS One. 2014 Apr 25;9(4):e96024. doi: 10.1371/journal.pone.0096024 (PMC4000202; doi:10.1371/journal.pone.0096024)

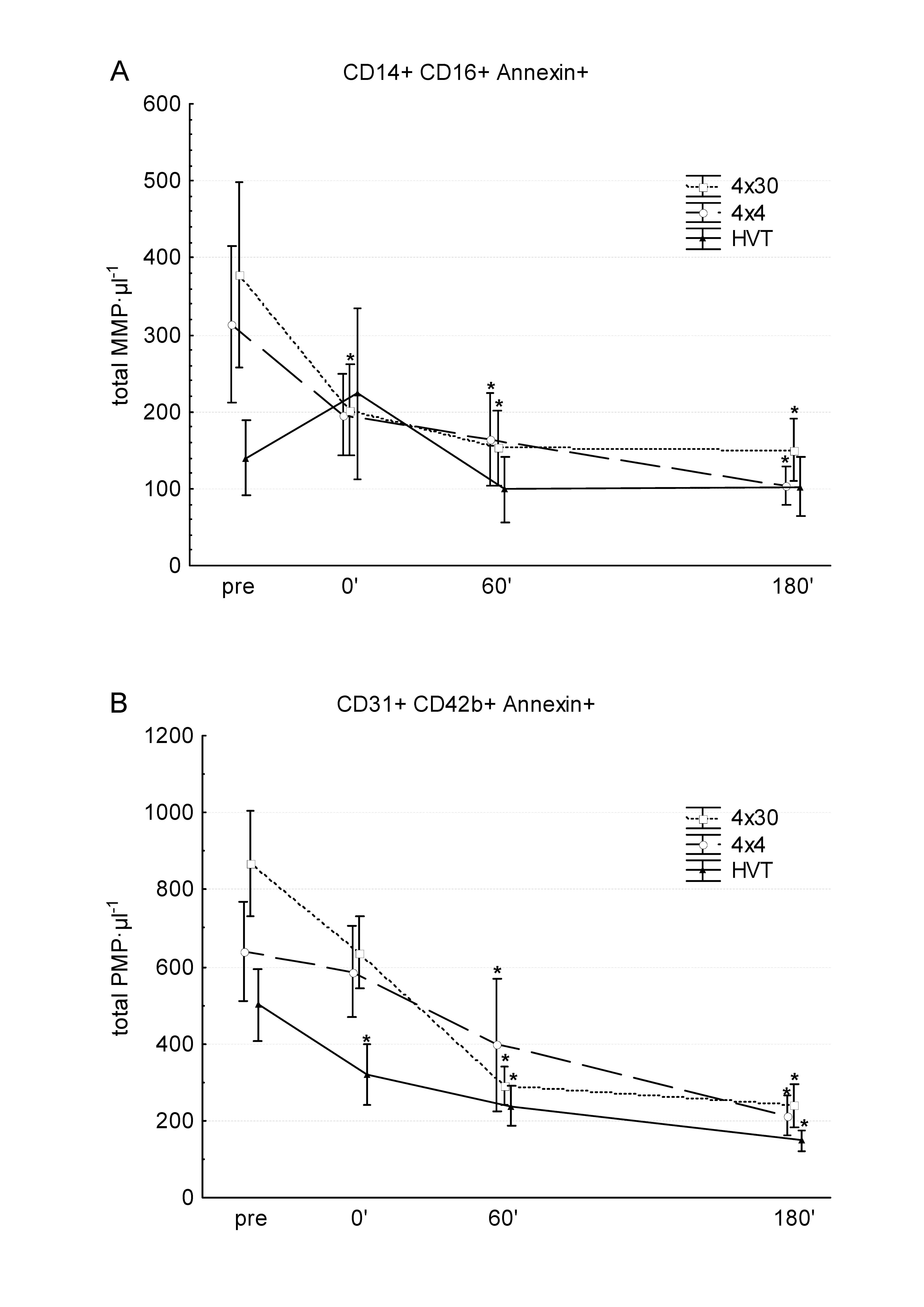

Supplement: Figure S1 — Changes in circulating MMP and PMP in conditioned serum of athletes. 4×30 sec (Squares, dotted line), 4×4 min (circles, broken line) and HVT (triangles, solid line). * significantly different compared to pre-values (p≤0.05). Values are presented as means ± SD. (TIFF) [file pone.0096024.s001.tiff]

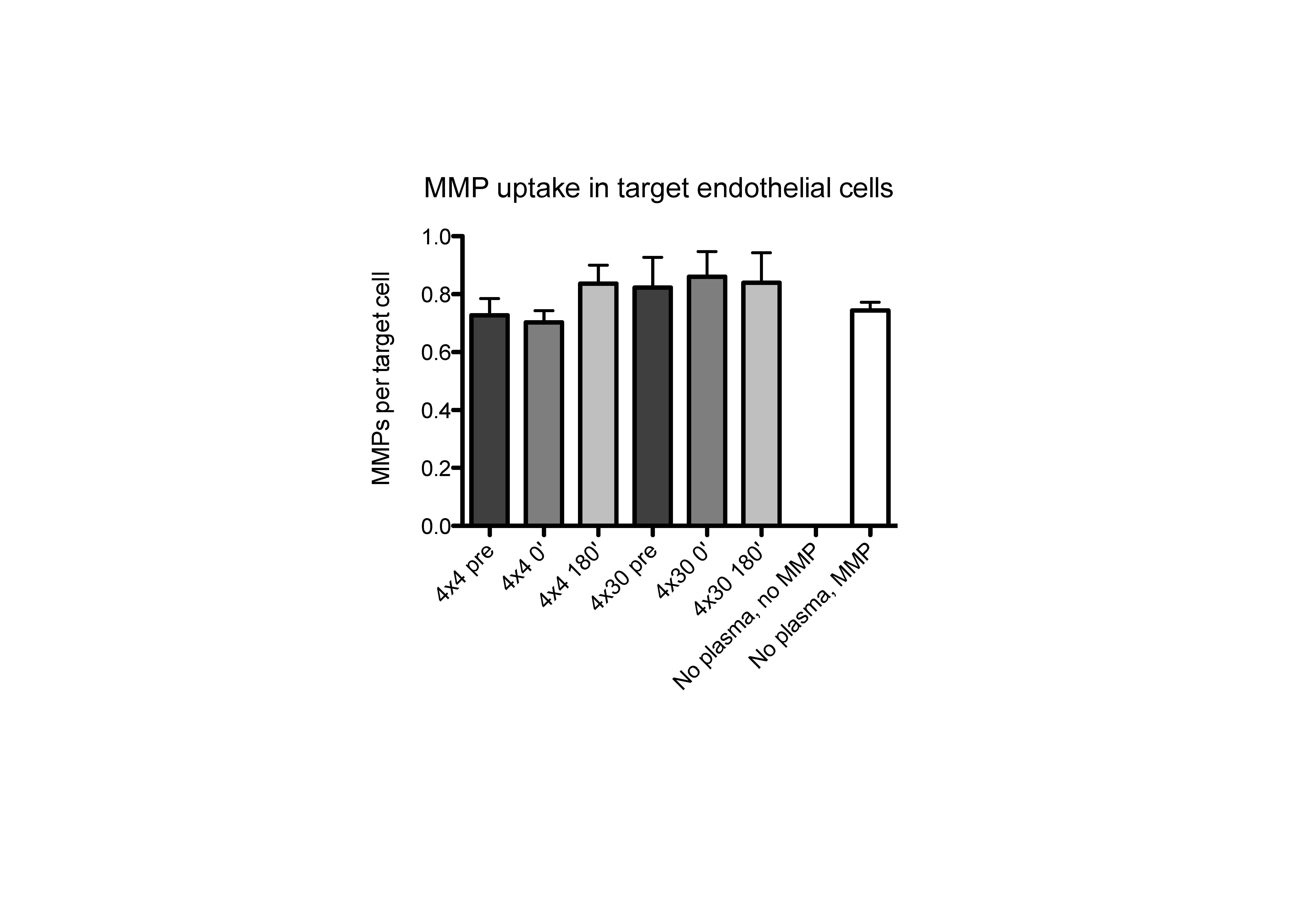

Supplement: Figure S2 — Uptake of MMP into target endothelial cells. HCAEC were stimulated with MMP and sera for 4 hours. N>3. (TIFF) [file pone.0096024.s002.tiff]

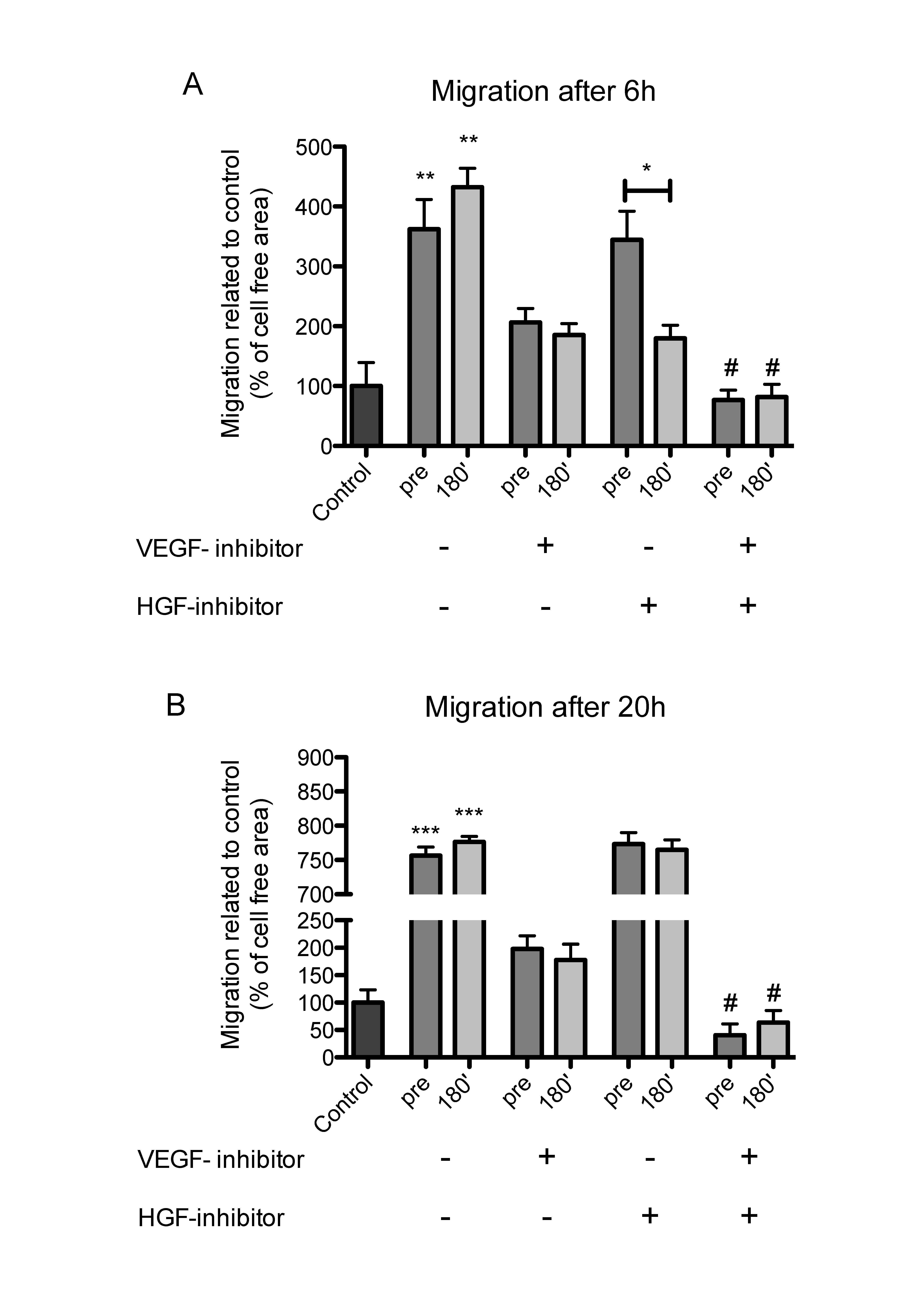

Supplement: Figure S3 — Migration of endothelial cells after stimulation with conditioned sera. HCAEC were stimulated with sera and with our without VEGF and HGF inhibitors. HAEC pretreated with growth factor deprived medium served as control. Migration was assessed after six (A) and twenty (B) hours. N = 5. A: ** = p<0.01 vs. Control, pre/180′ + VEGF-inhibitor and 180′ + HGF-inhibitor, # = p<0.001 vs. all other groups except control. B: *** = p<0.001 vs. Control and pre/180′ + VEGF-inhibitor, # = p<0.001 vs. all other groups except control. (TIFF) [file pone.0096024.s003.tiff]
